# Supplementary material for: State-of-the-Art Organ-on-Chip Models and Designs for Medical Applications: A Systematic Review
Source: Biomimetics (Basel). 2025 Aug 11;10(8):524. doi: 10.3390/biomimetics10080524 (PMC12383757; doi:10.3390/biomimetics10080524)
Supplement: Supplementary file 1 [file biomimetics-10-00524-s001.zip › Supplementary Material 01 _ String.pdf]

STRING

(“Organ on a chip”) OR (“Lab-On-A-Chip Device*”) OR (“Device” AND “Lab-On-A-Chip”) OR (“Devices” AND “Lab-On-A-Chip”) OR (“Microchip Analytical Device*”) OR (“Analytical Device*” AND “Microchip”) OR (“Device*” AND “Microchip Analytical”) OR (“Microfluidic Lab-On-A-Chip”) OR (“Lab-On-A-Chip*” AND “Microfluidic”) OR (“Microfluidic Lab On A Chip”) OR (“Microfluidic Lab-On-A-Chips”) OR (“Microfluidic Device*”) OR (“Device*” AND “Microfluidic”) OR (“Microfluidic Microchip*”) OR (“Microchip*” AND “Microfluidic”) OR (“Nanochip Analytical Devices”) OR (“Analytical Device*” AND “Nanochip”) OR (“Device*” AND “Nanochip Analytical”) OR (“Nanochip Analytical Device”) OR (“lab on a chip”) OR (“Body on a chip”) OR (“Microfluidic*”) OR (“micro-fluidics”) AND (“Cell Culture Techniques” AND “Three Dimensional”) OR (“3 D Cell Culture*”) OR (“3 Dimensional Cell Culture”) OR (“3-D Cell Culture*”) OR (“3-Dimensional Cell Culture*”) OR (“Culture*” AND “3-D Cell”) OR (“three dimensional cell culture”) OR (“t3-D cell culture”) OR (“3D cell culture technique”) AND (“Patient-Specific Modeling”) OR (“Computational Modeling” AND “Patient-Specific”) OR (“Modeling” AND “Patient-Specific”) OR (“Modeling” AND “Patient-Specific”) OR (“Patient Specific Modeling”) OR (“Patient-Specific Computational”) OR (“Physiome*”) OR (“computer aided design”) OR (“computer assisted design”) OR (“computer-aided design”) OR (“design” AND “computer assisted”) OR (“Cellular Microenvironment*”) OR (“Cell Microenvironment*”) OR (“Microenvironment*” AND “Cell*”) OR (“Computer Simulation*”) OR (“Computational Modeling”) OR (“Computational Modelling”) OR

(“Computer Model*”) OR (“Computerized Model*”) OR (“In silico Modeling”) OR (“In silico Model*”) OR (“In silico Simulation”) OR (“Model*” AND “Computer”) OR (“Model*” AND “Computerized”) OR (“In silico Simulation”) OR (“computer-based simulation”) AND Bioprinting OR (“Computer*Aided Design*”) OR (“CAD-CAM”) OR (“Computer*Aided Manufacturing”) OR (“Computer*Assisted Design*”) OR (“Computer*Assisted Manufacturing”) OR (“Computer*Aided Manufacturing”) OR (“Design*” AND “Computer-Aided”) OR (“Design*” “Computer-Assisted”) OR (“Manufacturing” AND “Computer-Aided”) OR (“Manufacturing” AND “Computer-Assisted”) OR (“Equipment Design*”) OR (“Design*” AND “Device*”) OR (“Design*” AND “Equipment ”) OR (“Design*” AND “Medical Device”) OR (“Device Design*” AND “Medical”) AND (“Biomedical Technolog*”) OR (“Health Care Technology”) OR (“Health Technology”) OR (“Medical Technology”) OR (“Technology” AND “Biomedical”) OR (“Technology” AND “Health”) OR (“Technology” AND “Health Care”) OR (“bio*medical technology”) OR (“medical lab science”) OR (“medical lab technology”) OR (“medical laboratory science”) OR (“medical laboratory technology”) OR (“medical research technology”) OR (“stains and staining”) OR (“technology” AND “medical”) OR (“technology” AND “medical laboratory”)
