# Supplementary material for: State-of-the-Art Organ-on-Chip Models and Designs for Medical Applications: A Systematic Review
Source: Biomimetics (Basel). 2025 Aug 11;10(8):524. doi: 10.3390/biomimetics10080524 (PMC12383757; doi:10.3390/biomimetics10080524)
Supplement: Supplementary file 1 [file biomimetics-10-00524-s001.zip › Supplementary Material 02_ Database search and results.pdf]

Database search and results

Database	Search	Quantity of Publications
**Scopus**<br>January 25, 2023<br>Update: June 04, 2025	((TITLE-ABS-KEY (("Organ on a chip"))) OR (TITLE-ABS-KEY (("Lab-On-A-Chip Device*") OR ("Device" AND "Lab-On-A-Chip") OR ("Devices" AND "Lab-On-A-Chip") OR ("Microchip Analytical Device*") OR ("Analytical Device*" AND "Microchip") OR ("Device*" AND "Microchip Analytical") OR ("Microfluidic Lab-On-A-Chip") OR ("Lab-On-A-Chip*" AND "Microfluidic") OR ("Microfluidic Lab On A Chip") OR ("Microfluidic Lab-On-A-Chips") OR ("Microfluidic Device*") OR ("Device*" AND "Microfluidic") OR ("Microfluidic Microchip*") OR ("Microchip*" AND "Microfluidic") OR ("Nanochip Analytical Devices") OR ("Analytical Device*" AND "Nanochip") OR ("Device*" AND "Nanochip Analytical") OR ("Nanochip Analytical Device"))) OR (TITLE-ABS-KEY (("lab on a chip"))) OR (TITLE-ABS-KEY (("Body on a chip"))) OR (TITLE-ABS-KEY (("Microfluidic*") OR ("micro-fluidics"))))) AND (TITLE-ABS-KEY (("Cell Culture Techniques" AND "Three Dimensional") OR ("3 D Cell Culture*") OR ("3 Dimensional Cell Culture") OR ("3-D Cell Culture*") OR ("3-Dimensional Cell Culture*") OR ("Culture*" AND "3-D Cell") OR ("three dimensional cell culture") OR ("t3-D cell culture") OR ("3D cell culture technique"))) AND ((TITLE-ABS-KEY (("Patient-Specific Modeling") OR ("Computational Modeling" AND "Patient-Specific") OR ("Modeling" AND "Patient-Specific") OR ("Modeling" AND "Patient-Specific") OR ("Patient Specific Modeling") OR ("Patient-Specific Computational") OR ("Physiome*") OR ("computer aided design") OR ("computer assisted design") OR ("computer-aided design") OR ("design" AND "computer assisted"))) OR (TITLE-ABS-KEY (("Cellular Microenvironment*") OR ("Cell Microenvironment*") OR ("Microenvironment*" AND "Cell*"))) OR (TITLE-ABS-KEY (("Computer Simulation*") OR ("Computational Modeling") OR ("Computational Modelling") OR ("Computer Model*") OR ("Computerized Model*") OR ("In silico Modeling") OR ("In silico Model*") OR ("In silico Simulation") OR ("Model*" AND "Computer") OR ("Model*" AND "Computerized") OR ("In silico Simulation") OR ("computer-based simulation"))))) AND ((TITLE-ABS-KEY (bioprinting)) OR (TITLE-ABS-KEY (("Computer*Aided Design*") OR ("CAD-CAM") OR ("Computer*Aided Manufacturing") OR ("Computer*Assisted Design*") OR ("Computer*Assisted Manufacturing") OR ("Computer*Aided Manufacturing") OR ("Design*" AND "Computer-Aided") OR ("Design*" "Computer-Assisted") OR ("Manufacturing" AND "Computer-Aided") OR ("Manufacturing" AND "Computer-Assisted"))) OR (TITLE-ABS-KEY (("Equipment Design*") OR ("Design*" AND "Device*") OR ("Design*" AND "Equipment ") OR ("Design*" AND "Medical Device") OR ("Device Design*" AND "Medical")))) AND (TITLE-ABS-KEY (("Biomedical Technolog*") OR ("Health Care Technology") OR ("Health Technology") OR ("Medical Technology") OR ("Technology" AND "Biomedical") OR ("Technology" AND "Health") OR ("Technology" AND "Health Care") OR ("bio*medical technology") OR ("medical lab science") OR ("medical lab technology") OR ("medical laboratory science") OR ("medical laboratory technology") OR ("medical research technology") OR ("stains and staining") OR ("technology" AND "medical") OR ("technology" AND "medical laboratory")))	**Total:** 23<br><br>**Total after applying the filter:** 21
**Pubmed**<br>January 25, 2023<br>Update: June 04, 2025	("Microphysiological Systems"[MeSH Terms] OR "Lab-On-A-Chip Devices"[MeSH Terms] OR "Microfluidics"[MeSH Terms]) AND "cell culture techniques, three dimensional"[MeSH Terms] AND ("Patient-Specific Modeling"[MeSH Terms] OR "Cellular Microenvironment"[MeSH Terms] OR "Computer Simulation"[MeSH Terms]) AND ("Bioprinting"[MeSH Terms] OR "Computer-Aided Design"[MeSH Terms] OR "Equipment Design"[MeSH Terms]) AND "Biomedical Technology"[MeSH Terms]	**Total:** 2<br><br>**Total after applying the filter:** 0
**Embase**<br>January 25, 2023<br>Update: June 04, 2025	'three dimensional cell culture'/exp AND 'medical technology'/exp AND ('organ on a chip'/exp OR 'lab on a chip'/exp OR 'body on a chip'/exp OR 'microfluidics'/exp) AND ('biological model'/exp OR 'computer simulation'/exp) AND ('bioprinting'/exp OR 'equipment design'/exp)	**Total:** 647<br><br>**Total after applying the filter:** 644
**Web of Science**<br>January 25, 2023	AB = ("Organ on a chip") OR ("Lab-On-A-Chip Device*") OR ("Device" AND "Lab-On-A-Chip") OR ("Devices" AND "Lab-On-A-Chip") OR ("Microchip Analytical Device*") OR ("Analytical Device*" AND "Microchip") OR ("Device*" AND "Microchip Analytical") OR ("Microfluidic Lab-On-A-Chip") OR ("Lab-On-A-Chip*" AND "Microfluidic") OR ("Microfluidic Lab On A Chip") OR ("Microfluidic Lab-On-A-Chips") OR ("Microfluidic Device*") OR ("Device*" AND "Microfluidic") OR ("Microfluidic Microchip*") OR ("Microchip*" AND "Microfluidic") OR ("Nanochip Analytical Devices") OR ("Analytical Device*" AND "Nanochip") OR ("Device*" AND "Nanochip Analytical") OR ("Nanochip Analytical Device") OR ("lab on a chip") OR ("Body on a chip") OR ("Microfluidic*") OR ("micro-fluidics") AND ("Cell Culture Techniques" AND "Three Dimensional") OR ("3 D Cell Culture*") OR ("3 Dimensional Cell Culture") OR ("3-D Cell Culture*") OR ("3-Dimensional Cell Culture*") OR ("Culture*" AND "3-D Cell") OR ("three dimensional cell culture") OR ("t3-D cell culture") OR ("3D cell culture technique") AND ("Patient-Specific Modeling") OR ("Computational Modeling" AND "Patient-Specific") OR ("Modeling" AND "Patient-Specific") OR ("Modeling" AND "Patient-Specific") OR ("Patient Specific Modeling") OR ("Patient-Specific Computational") OR ("Physiome*") OR ("computer aided design") OR ("computer assisted design") OR ("computer-aided design") OR ("design" AND "computer assisted") OR ("Cellular Microenvironment*") OR ("Cell Microenvironment*") OR ("Microenvironment*" AND "Cell*") OR ("Computer Simulation*") OR ("Computational Modeling") OR ("Computational Modelling") OR ("Computer Model*") OR ("Computerized Model*") OR ("In silico Modeling") OR ("In silico Model*") OR ("In silico Simulation") OR ("Model*" AND "Computer") OR ("Model*" AND "Computerized") OR ("In silico Simulation") OR ("computer-based simulation") AND Bioprinting OR	**Total:** 05<br><br>**Total after applying the filter:** 03

Update: June 04, 2025	(“Computer*Aided Design*”) OR (“CAD-CAM”) OR (“Computer*Aided Manufacturing”) OR (“Computer*Assisted Design*”) OR (“Computer*Assisted Manufacturing”) OR (“Computer*Aided Manufacturing”) OR (“Design*” AND “Computer-Aided”) OR (“Design*” “Computer-Assisted”) OR (“Manufacturing” AND “Computer-Aided”) OR (“Manufacturing” AND “Computer-Assisted”) OR (“Equipment Design*”) OR (“Design*” AND “Device*”) OR (“Design*” AND “Equipment ”) OR (“Design*” AND “Medical Device”) OR (“Device Design*” AND “Medical”) AND (“Biomedical Technolog*”) OR (“Health Care Technology”) OR (“Health Technology”) OR (“Medical Technology”) OR (“Technology” AND “Biomedical”) OR (“Technology” AND “Health”) OR (“Technology” AND “Health Care”) OR (“bio*medical technology”) OR (“medical lab science”) OR (“medical lab technology”) OR (“medical laboratory science”) OR (“medical laboratory technology”) OR (“medical research technology”) OR (“stains and staining”) OR (“technology” AND “medical”) OR (“technology” AND “medical laboratory”)	
**IEEE Xplore** January 25, 2023 June 04, 2025	("Abstract":Organ on a chip) OR ("Abstract":Lab-On-A-Chip Devices) OR ("Abstract":Body on a chip) OR ("Abstract":Microfluidics) AND ("Abstract":Cell Culture Techniques, Three Dimensional) AND ("Abstract":Patient-Specific Modeling) OR ("Abstract":Cellular Microenviroment) OR ("Abstract":Computer Simulation) AND ("Abstract":Bioprinting) OR ("Abstract":Computer-Aided Design) AND ("Abstract":Biomedical Technology)	**Total:** 2728 **Total after applying the filter:** 1372
**CINAHL/ EBSCO** January 25, 2023 June 04, 2025	(“Organ on a chip”) OR (“Lab-On-A-Chip Device*”) OR (“Device” AND “Lab-On-A-Chip”) OR (“Devices” AND “Lab-On-A-Chip”) OR (“Microchip Analytical Device*”) OR (“Analytical Device*” AND “Microchip”) OR (“Device*” AND “Microchip Analytical”) OR (“Microfluidic Lab-On-A-Chip”) OR (“Lab-On-A-Chip*” AND “Microfluidic”) OR (“Microfluidic Lab On A Chip”) OR (“Microfluidic Lab-On-A-Chips”) OR (“Microfluidic Device*”) OR (“Device*” AND “Microfluidic”) OR (“Microfluidic Microchip*”) OR (“Microchip*” AND “Microfluidic”) OR (“Nanochip Analytical Devices”) OR (“Analytical Device*” AND “Nanochip”) OR (“Device*” AND “Nanochip Analytical”) OR (“Nanochip Analytical Device”) OR (“lab on a chip”) OR (“Body on a chip”) OR (“Microfluidic*”) OR (“micro-fluidics”) AND (“Cell Culture Techniques” AND “Three Dimensional”) OR (“3 D Cell Culture*”) OR (“3 Dimensional Cell Culture”) OR (“3-D Cell Culture*”) OR (“3-Dimensional Cell Culture*”) OR (“Culture*” AND “3-D Cell”) OR (“three dimensional cell culture”) OR (“t3-D cell culture”) OR (“3D cell culture technique”) AND (“Patient-Specific Modeling”) OR (“Computational Modeling” AND “Patient-Specific”) OR (“Modeling” AND “Patient-Specific”) OR (“Modeling” AND “Patient-Specific”) OR (“Patient Specific Modeling”) OR (“Patient-Specific Computational”) OR (“Physiome*”) OR (“computer aided design”) OR (“computer assisted design”) OR (“computer-aided design”) OR (“design” AND “computer assisted”) OR (“Cellular Microenvironment*”) OR (“Cell Microenvironment*”) OR (“Microenvironment*” AND “Cell*”) OR (“Computer Simulation*”) OR (“Computational Modeling”) OR (“Computational Modelling”) OR (“Computer Model*”) OR (“Computerized Model*”) OR (“In silico Modeling”) OR (“In silico Model*”) OR (“In silico Simulation”) OR (“Model*” AND “Computer”) OR (“Model*” AND “Computerized”) OR (“In silico Simulation”) OR (“computer-based simulation”) AND Bioprinting OR (“Computer*Aided Design*”) OR (“CAD-CAM”) OR (“Computer*Aided Manufacturing”) OR (“Computer*Assisted Design*”) OR (“Computer*Assisted Manufacturing”) OR (“Computer*Aided Manufacturing”) OR (“Design*” AND “Computer-Aided”) OR (“Design*” “Computer-Assisted”) OR (“Manufacturing” AND “Computer-Aided”) OR (“Manufacturing” AND “Computer-Assisted”) OR (“Equipment Design*”) OR (“Design*” AND “Device*”) OR (“Design*” AND “Equipment ”) OR (“Design*” AND “Medical Device”) OR (“Device Design*” AND “Medical”) AND (“Biomedical Technolog*”) OR (“Health Care Technology”) OR (“Health Technology”) OR (“Medical Technology”) OR (“Technology” AND “Biomedical”) OR (“Technology” AND “Health”) OR (“Technology” AND “Health Care”) OR (“bio*medical technology”) OR (“medical lab science”) OR (“medical lab technology”) OR (“medical laboratory science”) OR (“medical laboratory technology”) OR (“medical research technology”) OR (“stains and staining”) OR (“technology” AND “medical”) OR (“technology” AND “medical laboratory”)	**Total:** 6.382 **Total after applying the filter:** 3765
	**Total number of articles found in the databases:**	**9790**
	**Total number of articles found after applying the filters and removing duplicates:**	**3150**
	**Filters used**: research from the last ten years (2013-2023) and types of articles	
