# Supplementary material for: State-of-the-Art Organ-on-Chip Models and Designs for Medical Applications: A Systematic Review
Source: Biomimetics (Basel). 2025 Aug 11;10(8):524. doi: 10.3390/biomimetics10080524 (PMC12383757; doi:10.3390/biomimetics10080524)
Supplement: Supplementary file 1 [file biomimetics-10-00524-s001.zip › Supplementary Material 03 _ Excluded Articles and Reasons for Exclusion.pdf]

Excluded Articles and Reasons for Exclusion

Reasons for exclusion	Total
**Reason 01.** Conferences	04
**Reason 02.** Review articles	19
**Reason 03.** Articles not related to 3D cell culture or microfluidics	07
**Reason 04.** 2D in vitro studies or purely in silico	12
**Reason 05.** Work related to biosensors	15
**Reason 06.** Articles that do not present the device model, architecture, functionality, or biomaterials for the construction of Organs-on-a-chip	35
**Articles excluded after full-text reading:**	**92**
