# Supplementary material for: State-of-the-Art Organ-on-Chip Models and Designs for Medical Applications: A Systematic Review
Source: Biomimetics (Basel). 2025 Aug 11;10(8):524. doi: 10.3390/biomimetics10080524 (PMC12383757; doi:10.3390/biomimetics10080524)
Supplement: Supplementary file 1 [file biomimetics-10-00524-s001.zip › Supplementary Material 04_Risk of Bias_questions.pdf]

Supplementary Material

Table Risk of bias of selected individual studies

---

1. **Does the study clearly describe its research objectives and hypotheses before initiating the development of the new Organ-on-a-Chip platform model?**
2. **Does the study refer to previous studies or models that may have influenced the development of the new Organ-on-a-Chip platform model? Are these references presented objectively and impartially?**
3. **Were efforts made to compare the new Organ-on-a-Chip platform model with existing models to ensure an adequate analysis of advantages and disadvantages?**
4. **Does the study provide detailed information about the development process of the new Organ-on-a-Chip platform model, including all steps, methods, and printing criteria used?**
5. **Were experiments conducted with treatment and control groups for the analysis and comparison of culture media?**
6. **Was the study submitted to a Research Ethics Committee appropriate to the type of cell culture used?**
7. **Was there any bias in the selection of parameters or conditions used in the microfluidic analysis of the Organ-on-a-Chip platform? Is there a risk that only the most favorable or convenient results were presented while other relevant data were omitted?**
8. **Are there any declared conflicts of interest by the study's authors that may affect the interpretation of results or the disclosure of information about the new Organ-on-a-Chip platform model?**
9. **Was the result analysis conducted in a blinded manner, so that reviewers or researchers involved had no prior knowledge of the samples or experimental conditions to avoid performance bias?**
10. **Were measures taken to ensure that data collection for the new Organ-on-a-Chip platform model was standardized and replicable across all stages of the study?**
11. **Did the study consider possible confounding factors that could influence the results of the new Organ-on-a-Chip platform model? Were adequate measures taken to control or adjust for these factors in the analysis?**
12. **Are the presented results based on an adequate number of repetitions or replicates to ensure robustness and reliability of the findings?**
13. **Did the study transparently address any challenges or problems encountered during the development of the new Organ-on-a-Chip platform model? Were potential solutions or improvements presented?**
